# Severe clinical spectrum with high mortality in pediatric patients with COVID-19 and multisystem inflammatory syndrome

**DOI:** 10.6061/clinics/2020/e2209

**Published:** 2020-08-14

**Authors:** Maria Fernanda Badue Pereira, Nadia Litvinov, Sylvia Costa Lima Farhat, Adriana Pasmanik Eisencraft, Maria Augusta Bento Cicaroni Gibelli, Werther Brunow de Carvalho, Vinicius Rodrigues Fernandes, Thais de Toledo Fink, Juliana Valéria de Souza Framil, Karine Vusberg Galleti, Alice Lima Fante, Maria Fernanda Mota Fonseca, Andreia Watanabe, Camila Sanson Yoshino de Paula, Giovanna Gavros Palandri, Gabriela Nunes Leal, Maria de Fatima Rodrigues Diniz, João Renato Rebello Pinho, Clovis Artur Silva, Heloisa Helena de Sousa Marques, Pediatric COVID HC-FMUSP Study Group, Alfio Rossi Junior, Artur Figueiredo Delgado, Anarella Penha Meirelles de Andrade, Claudio Schvartsman, Ester Cerdeira Sabino, Mussya Cisotto Rocha, Kelly Aparecida Kanunfre, Thelma Suely Okay, Magda Maria Sales Carneiro-Sampaio, Patricia Palmeira Daenekas Jorge

**Affiliations:** I Instituto da Crianca e do Adolescente (ICr), Hospital das Clinicas HCFMUSP, Faculdade de Medicina, Universidade de Sao Paulo, Sao Paulo, SP, BR.; II Faculdade Medicina FMUSP, Universidade de Sao Paulo, Sao Paulo, SP, BR.; III Hospital das Clinicas HCFMUSP, Faculdade de Medicina, Universidade de Sao Paulo, Sao Paulo, SP, BR.; IV Instituto de Tratamento do Cancer Infantil, Hospital das Clinicas HCFMUSP, Faculdade de Medicina, Universidade de Sao Paulo, Sao Paulo, SP, BR.; V Laboratorio de Biologia Molecular, Hospital das Clinicas HCFMUSP, Faculdade de Medicina, Universidade de Sao Paulo, Sao Paulo, SP, BR.

**Keywords:** COVID-19, Children, Adolescent, Outcome, Immunosuppression, Multisystem Inflammatory Syndrome

## Abstract

**OBJECTIVES:**

To assess the outcomes of pediatric patients with laboratory-confirmed coronavirus disease (COVID-19) with or without multisystem inflammatory syndrome in children (MIS-C).

**METHODS:**

This cross-sectional study included 471 samples collected from 371 patients (age<18 years) suspected of having severe acute respiratory syndrome coronavirus 2 (SARS-CoV-2) infection. The study group comprised 66/371 (18%) laboratory-confirmed pediatric COVID-19 patients: 61 (92.5%) patients tested positive on real-time reverse transcription-polymerase chain reaction tests for SARS-CoV-2, and 5 (7.5%) patients tested positive on serological tests. MIS-C was diagnosed according to the criteria of the Center for Disease Control.

**RESULTS:**

MIS-C was diagnosed in 6/66 (9%) patients. The frequencies of diarrhea, vomiting, and/or abdominal pain (67% *vs.* 22%, *p*=0.034); pediatric SARS (67% *vs.* 13%, *p*=0.008); hypoxemia (83% *vs.* 23%, *p*=0.006); and arterial hypotension (50% *vs.* 3%, *p*=0.004) were significantly higher in patients with MIS-C than in those without MIS-C. The frequencies of C-reactive protein levels >50 mg/L (83% *vs.* 25%, *p*=0.008) and D-dimer levels >1000 ng/mL (100% *vs.* 40%, *p*=0.007) and the median D-dimer, troponin T, and ferritin levels (*p*<0.05) were significantly higher in patients with MIS-C. The frequencies of pediatric intensive care unit admission (100% *vs.* 60%, *p*=0.003), mechanical ventilation (83% *vs.* 7%, *p*<0.001), vasoactive agent use (83% *vs.* 3%, *p*<0.001), shock (83% *vs.* 5%, *p*<0.001), cardiac abnormalities (100% *vs.* 2%, *p*<0.001), and death (67% *vs.* 3%, *p*<0.001) were also significantly higher in patients with MIS-C. Similarly, the frequencies of oxygen therapy (100% *vs.* 33%, *p*=0.003), intravenous immunoglobulin therapy (67% *vs.* 2%, *p*<0.001), aspirin therapy (50% *vs.* 0%, *p*<0.001), and current acute renal replacement therapy (50% *vs.* 2%, *p*=0.002) were also significantly higher in patients with MIS-C. Logistic regression analysis showed that the presence of MIS-C was significantly associated with gastrointestinal manifestations [odds ratio (OR)=10.98; 95%CI (95% confidence interval)=1.20-100.86; *p*=0.034] and hypoxemia [OR=16.85; 95%CI=1.34-211.80; *p*=0.029]. Further univariate analysis showed a positive association between MIS-C and death [OR=58.00; 95%CI=6.39-526.79; *p*<0.0001].

**CONCLUSIONS:**

Pediatric patients with laboratory-confirmed COVID-19 with MIS-C had a severe clinical spectrum with a high mortality rate. Our study emphasizes the importance of investigating MIS-C in pediatric patients with COVID-19 presenting with gastrointestinal involvement and hypoxemia.

## INTRODUCTION

The novel coronavirus disease (COVID-19), caused by severe acute respiratory syndrome coronavirus 2 (SARS-CoV-2), has affected more than nine million people worldwide until June 26, 2020 (1,2).

This emerging infectious disease has been described mainly in adult patients. Indeed, studies conducted in China, Italy, and United States revealed that pediatric cases of laboratory-confirmed COVID-19 were rare, with incidences ranging from 1.2% to 2% (3-6). A recent systematic review revealed that, in contrast to adults with COVID-19, the vast majority of children and adolescents infected with SARS-CoV-2 had a milder disease course, with deaths rarely being reported. Severe and critical cases of COVID-19 included only 5% and 0.6% of pediatric cases, respectively. Approximately half of the critical COVID-19 cases were seen in infants less than 1 year old (3,7).

The clinical spectrum of pediatric COVID-19 is very wide, ranging from asymptomatic to critical illness (2,7-9). The most common signs and symptoms in children and adolescents are fever and cough, followed by headache, sore throat, myalgia, shortness of breath, nausea, abdominal pain, vomiting, and diarrhea (3,6,10). Other clinical features, such as renal, cutaneous, olfactory, gustatory, neurological, and ocular features, are infrequently reported in pediatric COVID-19 populations (3,8,10). Leucopenia, lymphopenia, and increased inflammatory markers are the most frequently reported laboratory abnormalities in these patients (2,3).

Nasopharyngeal swab samples are taken for real-time reverse transcription-polymerase chain reaction (real-time RT-PCR), as this technique is the gold standard for early diagnosis of SARS-CoV-2 infection. It is usually performed five to six days after the onset of signs/symptoms. Antibody testing is of relevance for the diagnosis of late infection and is generally performed after 14 days of infection (11,12).

Preexisting chronic diseases among pediatric patients with COVID-19 (6,13), particularly immunosuppressive illnesses, have been described in a small case series of patients hospitalized in general wards or pediatric intensive care units (PICU) (14,15). Recently, a new syndrome, called multisystem inflammatory syndrome in children (MIS-C), with clinical characteristics similar to those of Kawasaki disease (KD), KD shock syndrome, and macrophage-activating syndrome (MAS), has been reported in serious pediatric COVID-19 patients globally (2,16-19). However, to the best of our knowledge, an overall comparative evaluation of patients with and without MIS-C, including pediatric patients with preexisting chronic diseases and an immunocompromised status, has not been performed. Further, a systematic analysis of pediatric patients with laboratory-confirmed COVID-19 with and without MIS-C in a single Latin American tertiary and university hospital population has also not been performed.

Therefore, the objective of the present study was to assess demographic data, clinical manifestations, laboratory abnormalities, underlying conditions, outcomes, and treatments in pediatric patients with laboratory-confirmed COVID-19 with and without MIS-C.

## METHODS

A cross-sectional study was conducted between April 16, 2020 and June 21, 2020. During that period, 471 samples for SARS-CoV-2 detection were collected from 371 patients who were younger than 18 years. All 371 patients were suspected of having pediatric COVID-19 on the basis of the presence of at least one of the five warning signs. Our university hospital continuously updated the guidelines for warning signs during the COVID-19 pandemic. The warning signs included the following: 1) flu-like syndrome in high-risk children (<5 years old or with underlying conditions), 2) fever without a source, and/or 3) pediatric severe acute respiratory syndrome (SARS). Since April, 30, 2020, two additional definitions were added as warning signs for pediatric COVID-19: 4) KD (complete or incomplete), KD shock syndrome, or MAS (16,17) and 5) gastrointestinal signs/symptoms.

Pediatric COVID-19 was not confirmed in 305 children and adolescents. The study group comprised the remaining 66/371 (18%) pediatric patients with laboratory-confirmed COVID-19: 61 (92.5%) of the patients tested positive following real-time RT-PCR tests for SARS-CoV-2 and 5 (7.5%) of the patients tested positive following serological tests for SARS-CoV-2. All 66 pediatric patients with laboratory-confirmed COVID-19 were treated at the Instituto Central, Instituto da Criança e do Adolescente and Instituto de Tratamento do Cancer Infantil do Hospital das Clínicas da Faculdade de Medicina de São Paulo (HCFMUSP), São Paulo, Brazil. This study was approved by the ethics committee of our institution.

### Data collection of pediatric patients with laboratory-confirmed COVID-19 and study definitions

All data were recorded using a standardized electronic chart for pediatric COVID-19, including new specific criteria and definitions updated during the course of the pandemic. Demographic data included current age, duration of signs/symptoms before diagnosis, and sex. The following clinical manifestations were analyzed: presence and duration of fever, nasal discharge, dyspnea, sneezing, cough, sore throat, anosmia, pneumonia, headache, conjunctivitis, cutaneous rash, neurological symptoms, diarrhea, vomiting, abdominal pain, pediatric SARS, hypoxemia, and arterial hypotension.

Underlying conditions were categorized in previously healthy subjects and those with preexisting pediatric chronic diseases. Pediatric chronic diseases were those that had been present for more than three months, and the diagnosis of each disease was based on the physician’s scientific knowledge and using valid methods or instruments according to professional and/or specific diagnostic criteria (20-22). Neonates, children or adolescents with at least one of the following preexisting pediatric chronic diseases were considered immunocompromised: primary immunodeficiency, malignancy, chronic kidney disease (stages 1-5). Furthermore, those who had undergone solid organ or hematopoietic stem cell transplantation or with an autoimmune disease and who were using immunosuppressive agents were considered immunocompromised.

Laboratory abnormalities at diagnosis of pediatric COVID-19 were identified by assessing the hemoglobin concentration; leucocyte, lymphocyte, and thrombocyte counts; levels of inflammatory markers (C-reactive protein, fibrinogen, D-dimer, and ferritin); lactate dehydrogenase level; levels of aspartate and alanine aminotransferase; blood urea level; and levels of serum creatinine, triglycerides, creatinine phosphokinase (CK), and troponin T. Abnormalities in lung radiography, computed tomography, and echocardiography images were also evaluated. Multiplex real-time PCR was performed for the detection of 21 different respiratory viruses. The outcomes assessed were hospitalization, duration of hospitalization, PICU admission, need for mechanical ventilation, administration of vasoactive agents, development of shock and cardiac abnormalities, and death. The main treatments administered were oxygen therapy, antibiotics, oseltamivir, intravenous immunoglobulin, enoxaparin, aspirin, systemic glucocorticoids, and dialysis.

MIS-C was diagnosed according to the criteria provided by the Center for Disease Control (CDC), which include the following: current age <21 years; fever (>38.0°C for ≥24 hours or report of fever ≥24 hours); increased levels of inflammatory markers; severe disease needing hospital admission; multisystem organ involvement (at least two of the following: cardiac, renal, respiratory, hematologic, gastrointestinal, cutaneous, or neurological involvements); AND no alternative diagnoses; AND positive RT-PCR, serology, or antigen test for current or recent SARS-CoV-2 infection or exposure to a patient with suspected or confirmed COVID-19 within four weeks before the onset of the signs and symptoms (23). Flu-like syndrome was defined as the presence of fever associated with cough and/or sore throat and at least one of the following concomitant signs/symptoms: myalgia, headache, arthralgia, dyspnea, conjunctivitis, malaise, and loss of appetite. Pediatric SARS was defined on the basis of the presence of flu-like syndrome and at least one of the following: dyspnea, oxygen saturation below 95% in room air, or signs of respiratory distress. Gastrointestinal involvement was assessed on the basis of the presence of diarrhea, vomiting, and/or abdominal pain. Cardiac complications included myocardial dysfunction, myocarditis, pericarditis, and/or coronary-artery aneurism z-scores ≥2.5.

### Molecular and serological methods performed in pediatric patients with laboratory-confirmed COVID-19

Real-time RT-PCR was performed on the respiratory specimens at the Molecular Biology Laboratory of the Instituto Central of HCFMUSP to detect genes of SARS-CoV-2, as described by Drosten et al. (24). Antibody testing helped detect serum antibodies against S proteins from the coronavirus spikes. This serological method was conducted at the Immunology Laboratory of the Instituto Central of HCFMUSP using two different methods during the COVID-19 pandemic: an immunochromatography assay for the detection of SARS-Cov-2-specific IgM and IgG antibodies and an anti-SARS-CoV-2 enzyme-linked immunosorbent assay for the detection of IgG antibodies (25,26).

### Statistical analysis

The Mann-Whitney U test and Student’s t-test were used as appropriate for continuous variables, and the results are presented as median (minimum and maximum values) or mean±standard deviation. The Chi-square test and Fisher’s exact test were used for categorical variables, as appropriate. Logistic regression analysis was performed using MIS-C as the dependent variable and variables that presented a statistical significance level of *p*<0.2 in the univariate analyses as independent variables. Significance levels for all analyses were set at 5%.

## RESULTS

Six pediatric patients with laboratory-confirmed COVID-19 had MIS-C (9%). All of them had fever, increased levels of inflammatory markers, and severe pediatric COVID-19 requiring hospital admission. Multisystem disease occurred in all MIS-C patients, with at least two or more of the following organ or systems involved: cardiac (n=6), renal (n=4), respiratory (n=4), hematologic (n=4), gastrointestinal (n=4), dermatologic (n=0), and neurological (n=1). Myocardial dysfunction, myocarditis, pericarditis, and coronary-artery aneurism with z-scores ≥2.5 was observed in three, one, five, and three MIS-C patients, respectively. SARS-CoV-2 infection was confirmed by RT-PCR in five MIS-C patients and by antibody testing in one MIS-C patient. Incomplete KD was observed in 1/6 MIS-C patients and KD shock syndrome in 2/6 MIS-C patients. One of the MIS-C patients with focal segmental glomerulosclerosis, steroid-resistant nephrotic syndrome also had MAS confirmed using bone marrow aspirate, analysis of which showed proliferated and activated macrophages phagocytosing hematopoietic elements, characterized by erythrophagocytosis, thrombocyte phagocytosis, and leucocyte phagocytosis. Four out of six MIS-C patients died: one healthy adolescent, one patient with primary immunodeficiency with concomitant liver fibrosis, and two patients with refractory malignancies.

Table 1 presents demographic data, clinical manifestations, and underlying conditions of laboratory-confirmed pediatric COVID-19 in patients with MIS-C *versus* without MIS-C. The frequencies of cough (83% *vs.* 37%, *p*=0.038); diarrhea, vomiting, and/or abdominal pain (67% *vs.* 22%, *p*=0.034); pediatric SARS (67% *vs.* 13%, *p*=0.008); hypoxemia (83% *vs.* 23%, *p*=0.006); and arterial hypotension (50% *vs.* 3%, *p*=0.004) were significantly higher in MIS-C patients than in those without this syndrome. No differences in demographic data and underlying conditions were evidenced in both groups (Table 1).

Table 2 shows data on laboratory exams, outcomes, and treatment in pediatric patients with laboratory-confirmed COVID-19 with *versus* without MIS-C. The frequencies of CRP level >50 mg/L (83% *vs.* 25%, *p*=0.008) and D-dimer level >1000 ng/mL (100% *vs.* 40%, *p*=0.007) were significantly higher in patients with MIS-C than in those without this severe complication. The median of D-dimer [13,412 (1,286-86,900) *vs.* 1,208 (493-29,295) ng/mL, *p*=0.010] and ferritin levels [3,660 (469-35,976) *vs.* 3,295 (2,567-8,000) ng/mL, *p*=0.007] were significantly higher in patients with MIS-C. The frequencies of PICU admission (100% *vs.* 60%, *p*=0.003), the need for mechanical ventilation (83% *vs.* 7%, *p*<0.001), and the administration of vasoactive agents (83% *vs.* 3%, *p*<0.001) as well as the presence of shock (83% *vs.* 5%, p<0.001), cardiac abnormalities (100% *vs.* 2%, *p*<0.001), and death (67% *vs.* 3%, *p*<0.001) were also significantly higher in MIS-C patients.

Respiratory panel of viruses was also evaluated in 20 pediatric patients with laboratory-confirmed COVID-19. Rhinovirus was also detected in two infants with COVID-19, both without MIS-C. One of them had Edwards Syndrome and died because of respiratory complications. The other patient did not have any complication and, therefore, did not require hospitalization.

Regarding treatments, the frequencies of needing oxygen therapy (100% *vs.* 33%, *p*=0.003), intravenous immunoglobulin (67% *vs.* 2%, *p*<0.001), aspirin (50% *vs.* 0%, *p*<0.001), and current acute renal replacement therapy (50% *vs.* 2%, *p*=0.002) were significantly higher in patients with MIS-C than in those without (Table 2).

Logistic regression analysis included MIS-C as a dependent variable, and cough, gastrointestinal manifestations (vomiting, abdominal pain, and/or diarrhea), and hypoxemia as independent variables. Pediatric patients with laboratory-confirmed COVID-19 with MIS-C was significantly associated with gastrointestinal manifestations (vomiting, abdominal pain, and/or diarrhea) [odds ratio (OR) 10.98; 95% confidence interval (CI) 1.20-100.86; *p*=0.034] and hypoxemia [OR 16.85; 95% CI 1.34-211.80; *p*=0.029].

Further univariate analysis revealed association between death, as a dependent variable, and: hypoxemia [OR 16.43; 95% CI 1.77-152.60; *p*=0.014], pediatric SARS [OR 37.86; 95% CI 3.85-372.77; *p*=0.002], and MIS-C [OR 58.00; 95% CI 6.39-526.79; *p*<0.0001].

## DISCUSSION

We demonstrated that pediatric patients with laboratory-confirmed COVID-19 with MIS-C had a severe and acute clinical spectrum with a high mortality rate. Gastrointestinal manifestations and hypoxemia were factors associated with this life-threatening hyperinflammatory syndrome.

Our University Hospital is the largest public and teaching hospital complex in Latin America, with 2400 beds. Facing the spread of COVID-19 in Sao Paulo, the Crisis Management Committee designated the Central Institute as an unit for COVID-19 care (27). A medical staff was organized for pediatric and adult COVID-19 patients that required inpatient care. Importantly, a dynamic and integrated pediatric multidisciplinary and multiprofessional team was also set up, which included physicians and fellows with various subspecialties, led by the Pediatric Infectious Disease Unit, as well as nurses, psychologists, physiotherapists, social workers, and nutritionists. This team was designated to care for and perform specific studies involving neonates, children, and adolescents with pediatric COVID-19.

The main strengths of this study was the protocol used, which involved clinical, laboratory, and imaging techniques, and the outcomes, which were continually updated during the global pandemic. We also evaluated approximately 500 samples from patients with suspected SARS-CoV-2 infection in the emergency room, neonatal and general wards, and PICU of our university hospital using defined clinical criteria. The inclusion of confirmed pediatric COVID-19 patients with MIS-C was important because it reinforced the specificity of the temporal association between these two conditions. We also included pediatric patients with chronic diseases and COVID-19 who made up a unique subset, accounting for approximately three-fourth of the patient population. In fact, this subgroup of patients is infrequently reported upon in the literature, especially patients with immunosuppression. One limitation of this study was the small sample size, and this was because of the very low incidence of pediatric COVID-19 globally (3-6).

We extended the findings of previous studies that compared pediatric COVID-19 patients with and without MIS-C by showing that gastrointestinal involvement and hypoxemia are relevant factors associated with this subgroup. Indeed, abdominal pain, vomiting, and/or diarrhea were observed in 80-97% of MIS-C patients during diagnosis. This may help to identify this severe multisystemic disease during the ongoing pandemic (17,18,28,29). The most important differential diagnosis for this condition in pediatric patients was viral gastroenteritis. The presence of serious additional features such as hypoxemia, arterial hypotension, and shock, which reinforces MIS-C diagnosis, may help in early detection in daily clinical practice (28).

Importantly, a high mortality rate was observed in our MIS-C patients. The high frequency of preexisting chronic and immunocompromising diseases combined with the late MIS-C diagnosis seemed to contribute to the high mortality. In fact, these patients underwent multiple rounds of immunosuppressive therapies and exhibited major disease complexity and severity, an increased number of hospitalizations, and higher susceptibility to severe viral infectious diseases (20-22). In contrast to our results, the mortality rate among MIS-C patients with and without laboratory-confirmed COVID-19, reported in other studies was as high as 12.5% (17,18,28-31).

Interestingly, severe cardiovascular abnormalities leading to necessity of vasoactive and ventilatory support were commonly observed in our MIS-C patients. These features are infrequently observed in classical KD shock syndrome, which supports the notion that MIS-C is truly a syndrome that is different from KD and is a new clinical spectrum of pediatric COVID-19 (17). Another clinical spectrum of MIS-C observed in our patients was MAS, confirmed using bone marrow aspirates. MAS has also been described in autoimmune diseases, primary immunodeficiencies, and malignancies and may be difficult to differentiate from MIS-C in clinical practice, as observed in one of our patients.

We confirmed that MIS-C was associated with high levels of serum biomarkers, specifically an increase in C-reactive protein, D-dimer, troponin, and ferritin parameters, indicating inflammation. The high levels of these markers have also been associated with cytokine storm, multi-organ dysfunction syndrome, and poor outcome (9,16).

Anti-inflammatory drugs, particularly intravenous immunoglobulin and systemic glucocorticosteroids, were the main drugs used to treat our MIS-C patients, similar to that reported in other studies (17-19,29). Further studies will be necessary to clarify the immune pathogenesis of and possible specific treatments (such as anti-tumor necrosis factor, anti-interleukin-6, and anti-interleukin-1 blockers) (29,32) for this intriguing syndrome, particularly in immunocompromised MIS-C pediatric patients.

In conclusion, pediatric patients with laboratory-confirmed COVID-19 with MIS-C had a severe clinical spectrum with high mortality rate. Our study emphasizes the importance of investigating MIS-C in pediatric COVID-19 patients presenting with gastrointestinal involvement and hypoxemia.

### Pediatric COVID HC-FMUSP Study Group

**Alfio Rossi Junior, Artur Figueiredo Delgado, Anarella Penha Meirelles de Andrade, Claudio Schvartsman** (Instituto da Crianca e do Adolescente (ICr), Hospital das Clinicas HCFMUSP, Faculdade de Medicina, Universidade de Sao Paulo, Sao Paulo, SP, BR), **Ester Cerdeira Sabino, Mussya Cisotto Rocha, Kelly Aparecida Kanunfre, Thelma Suely Okay** (Instituto de Medicina Tropical de São Paulo, Universidade de São Paulo, São Paulo, SP, BR); **Magda Maria Sales Carneiro-Sampaio** (Faculdade Medicina FMUSP, Universidade de Sao Paulo, Sao Paulo, SP, BR) **Patricia Palmeira Daenekas Jorge** (Laboratorio de Investigacao Medica (LIM 36), Departamento de Pediatria, Hospital das Clinicas HCFMUSP, Faculdade de Medicina, Universidade de Sao Paulo, Sao Paulo, SP, BR).

## AUTHOR CONTRIBUTIONS

All the authors contributed substantially to the conception and design of the study and in the analysis and interpretation of the data. All authors revised the work critically and approved the final version.

## Figures and Tables

**Table 1:** Demographic data, clinical manifestations, and underlying conditions in pediatric patients with laboratory-confirmed COVID-19 with *versus* without MIS-C.

Variables of pediatric patients with laboratory-confirmed COVID-19	With MIS-C (n=6)	Without MIS-C (n=60)	*p*
**Demographic data**			
Current age, years	7.78 (0.01-17.62)	11.8 (0.86-13.62)	0.608
Age >10 years	4 (67)	25 (42)	0.392
Duration of signs/symptoms before diagnosis, days	6 (1-15)	2 (0-21)	0.095
Male sex	5 (83)	28 (47)	0.197
**Clinical manifestations**			
Fever	6 (100)	47 (78)	0.589
Duration of fever, days	4 (0-15)	1 (0-10)	0.224
Nasal discharge	2 (33)	26 (43)	1.000
Dyspnea	4 (67)	26 (43)	0.399
Sneezing	0 (0)	10 (17)	0.580
Cough	5 (83)	22 (37)	0.038
Anosmia, n=37	0 (0)	5 (15)	1.000
Pneumonia	3 (50)	15 (25)	0.333
Headache, n=55	0 (0)	11 (22)	0.330
Conjunctivitis, n=55	0 (0)	2 (4)	1.000
Cutaneous rash	0 (0)	1 (2)	1.000
Diarrhea, vomiting, and/or abdominal pain	4 (67)	13 (22)	0.034
Neurological (seizure)	1 (17)	0 (0)	0.091
Pediatric SARS	4 (67)	8 (13)	0.008
Hypoxemia	5 (83)	14 (23)	0.006
Arterial hypotension	3 (50)	2 (3)	0.004
**Underlying conditions**			
Previously healthy	1 (17)	10 (17)	1.000
Preexisting chronic diseases	5 (83)	50 (73)	1.000
Immunocompromising	4 (67)	28 (47)	0.420
Primary immunodeficiency	1 (17)	0 (0)	0.091
Previously underwent solid organ transplantation or HSCT	0 (0)	6 (10)	1.000
Malignancy	3 (50)	13 (22)	0.148
Chronic kidney disease (stages 1-5)	1 (17)	6 (10)	0.445
Autoimmune diseases	0 (0)	4 (7)	1.000
Immunosuppressive agent use	3 (50)	25 (42)	0.693

Results are presented as n (%), median (minimum-maximum values), or mean±standard deviation and n (%). COVID-19 - coronavirus disease 19, MIS-C - multisystem inflammatory syndrome in children, SARS - severe acute respiratory syndrome, HSCT - hematopoietic stem cell transplantation.

**Table 2:** Laboratory exams, outcomes, and treatment in pediatric patients with laboratory-confirmed COVID-19 with *versus* without MIS-C.

Variables of pediatric patients with laboratory-confirmed COVID-19	With MIS-C (n=6)	Without MIS-C (n=60)	*p*
**Hematological parameters**			
Hemoglobin, g/dL	10.5±1.01	11.1±2.05	0.528
Leucocyte count/mm^3^	9,660 (4,07-21,28)	6,795 (100-28,17)	0.103
Lymphocyte count/mm^3^	950 (410-2,980)	1,780 (100-20,270)	0.103
Thrombocyte count/mm^3^	172,167±125,4232	243,333±148,5148	0.262
**Inflammatory markers**			
C-reactive protein, mg/L, n=63	171.65 (29.47-407.2)	6.03 (0.3-272.18)	0.003
C-reactive protein >50 mg/L	5 (83)	14 (25)	0.008
Fibrinogen, mg/dL, n=36	303 (281-760)	565 (364-842)	0.121
D-dimer, ng/mL, n=50	13,412 (1,286-86,900)	1,208 (493-29,295)	0.010
D-dimer >1000 ng/mL	6 (100)	22 (40)	0.007
Ferritin, ng/mL, n=35	3,660 (469-35,976)	3,295 (2,567-8,000)	0.007
**Other exams**			
Lactate dehydrogenase, U/L, n=37	1,807 (280-4,476)	407 (294-1,638)	0.168
Aspartate aminotransferase, U/L, n=60	117 (13-2002)	41 (27-117)	0.278
Alanine aminotransferase, U/L, n=60	57 (5-560)	24.5 (7-495)	0.498
Blood urea, mg/dL, n=60	46 (23-133)	23 (8-36)	0.053
Serum creatinine, mg/dL, n=61	1.13 (0.17-4.2)	0.32 (0.27-0.49)	0.351
Triglycerides, mg/dL, n=13	168 (132-750)	163 (112-177)	0.143
CK, U/L, n=38	181 (67-329)	26 (13-37)	0.347
Troponin T, ng/mL, n=49	0.083 (0.01-0.290)	0.008 (0.003-3.000)	0.006
**Lung radiographic and CT imaging**			
Pulmonary X-ray abnormalities, n=51	5/6 (83)	25/45 (55)	0.380
Pulmonary CT abnormalities, n=23	3/4 (75)	15/19 (79)	1.000
**Outcomes**			
Hospitalization	6 (100)	42 (70)	0,178
Duration of hospitalization, days			
PICU admission	6 (100)	20 (60)	0,003
Mechanical ventilation	5 (83)	4 (7)	<0.001
Vasoactive agents	5 (83)	2 (3)	<0.001
Shock	5 (83)	3 (5)	<0.001
Cardiac abnormalities, n=35	6 (100)	1 (2)	<0.001
Death	4 (67)	2 (3)	<0.001
**Treatments**			
Oxygen therapy	6 (100)	20 (33)	0.003
Antibiotic, n=65	6 (100)	34 (58)	0.074
Oseltamivir, n=65	4 (67)	25 (42)	0.239
Intravenous immunoglobulin	4 (67)	1 (2)	<0.001
Enoxaparin	2 (33)	5 (8)	0.118
Aspirin	3 (50)	0 (0)	<0.001
Systemic glucocorticoid	2 (33)	10 (17)	0.298
Dialysis for acute renal replacement therapy	3 (50)	1 (2)	0.002

Results are presented as n (%), median (minimum-maximum values), or mean±standard deviation and n (%). COVID-19 - multisystem inflammatory syndrome in children, MIS-C - multisystem inflammatory syndrome in children, PICU - pediatric intensive care unit, CK - creatine phosphokinase, CT- computer tomography. Normal reference values: fibrinogen (200-393 mg/dL), D-dimer (<500 ng/mL), ferritin (36-391 ng/mL), lactate dehydrogenase (120-300 U/L), aspartate aminotransferase (<37 U/L), alanine aminotransferase (<41 U/L), blood urea (10-50 mg/dL), serum creatinine (<1.04 mg/dL), triglycerides (<90 mg/dL), CK (<190 U/L), and troponin (<0.014 ng/mL).
